# Valgus mechanism inducing a unique “terrible triad injury” pattern in the elbow: a detailed clinical and surgical analysis

**DOI:** 10.3389/fsurg.2025.1570509

**Published:** 2025-07-17

**Authors:** Yanmao Wang, Shiyang Yu, Jian Ding

**Affiliations:** Department of Orthopedics, Shanghai Sixth People’s Hospital Affiliated to Shanghai Jiao Tong University School of Medicine, Shanghai, China

**Keywords:** elbow, terrible triad injuries, fracture, dislocation, LUCL lesion

## Abstract

**Aims:**

The most accepted theory to explain the mechanism of “terrible triad injury (TTI)” of the elbow was proposed by O’Driscoll, describing it as a result of rotatory instability. However, a small subset of TTI cases appears to follow a different mechanism based on their clinical presentation. The aim of this study was to describe this injury pattern in detail and to suggest a treatment strategy that may lead to improved outcomes.

**Patients and methods:**

Cases of elbow dislocation treated between July 2017 and July 2019 were analyzed and identified as the valgus-type TTI through radiographs and surgical findings. Fractures and associated injuries were evaluated and compared with non-valgus TTIs. The current treatment method and prognosis were reviewed to formulate a preliminary feasible treatment plan.

**Results:**

Of 313 patients, 13 were diagnosed with valgus-type TTI. The mean age of these patients was 45.8 years, with the majority (84.6%) sustaining injury from low-energy trauma. No neurovascular injuries were observed. Three patients were treated non-operatively, while 10 underwent surgical treatment. In these 10 cases, coronoid avulsion of the medial collateral ligament (MCL) and continuity of the lateral ulnar collateral ligament were confirmed. Elbow function had a Mayo Elbow Performance Score of 98 and a Quick-Disabilities of the Arm, Shoulder, and Hand score of 6.38. No re-interventions were required after the initial treatment. Of the 13 patients, eight showed non-union of the MCL avulsion, though this did not affect stability. The remaining patients achieved radiographic union at an average of 10.7 weeks.

**Conclusion:**

Valgus-type TTI is a rare and distinct variant of TTI, but is less severe than the classic form. Surgeons should be aware of its associated injuries. The treatment strategy described here allows for targeted management of the injury's individual components, potentially reducing the risk of treatment failure.

## Introduction

“Terrible triad injury” (TTI) refers to an elbow dislocation associated with both radial head and coronoid fractures. The elbow structures should be repaired and stabilized to allow early mobilization; otherwise, the injury may result in significant morbidity for the patient and is associated with an increased risk of chronic instability, post-traumatic arthrosis, and poor functional outcomes.

Common classification systems for elbow trauma include those devised by Regan and Morrey, O’Driscoll, Mason, and the Arbeitsgemeinschaft für Osteosynthesefragen/Orthopaedic Trauma Association AO/OTA (1–4). The first two systems are widely used to evaluate TTI, particularly the O’Driscoll classification, which introduced several injury patterns attempting to explain the force mechanisms behind coronoid process fractures in complex elbow injuries—most commonly associated with posterolateral rotatory instability (PLRI) (2). However, a limitation of these systems is that, despite aiming to encompass all forms of elbow injuries, some fracture patterns still do not fit within them.

While the most accepted theory for explaining the mechanism of TTI of the elbow is based on rotatory instability, some cases propose a different mechanism based on the nature of the injury (5). TTI typically involves elbow dislocation accompanied by fractures of the radial head and coronoid process, leading to significant joint instability and poor functional outcomes if not properly treated (6–8). However, some clinical cases suggest that different mechanisms, such as those involving valgus force, may result in a distinct injury pattern that requires different management strategies. The hypothesis of this study is that a valgus mechanism can lead to a unique pattern of TTI, characterized by specific radiographic and surgical findings, which may necessitate tailored treatment strategies for optimal outcomes.

The aim of the present study was to describe this injury pattern in detail and to suggest treatment strategies that may allow for improved management. We hypothesize that this atypical valgus TTI will demonstrate distinct, recurrent injury characteristics.

## Materials and methods

Using a prospectively collected orthopedic trauma database, we investigate all elbow dislocation cases treated at our hospital between July 2017 and July 2019. The diagnosis of TTI was confirmed based on the presence of its three components: radial head fracture, coronoid fracture, and elbow dislocation. Exclusion criteria included absence of computed tomography (CT) data, lack of detailed medical records or magnetic resonance imaging (MRI) to assess the integrity of the lateral ulnar collateral ligament (LUCL), follow-up <12 months, and skeletally immature patients. Patients aged over 80 years were also excluded to minimize potential confounding effects related to age-associated bone fragility, which may influence fracture healing and treatment outcomes. This age threshold was based on clinical practice and existing guidelines, acknowledging that elderly individuals aged over 80 years typically present with poorer bone quality. We recognize this cutoff is somewhat arbitrary, and future studies could examine the effects of bone quality across a broader age range.

The study was approved by the hospital’s ethics committee.

### Diagnosis

Valgus-type TTI was identified mainly through radiographs, CT, and MRI. Plain radiographs were examined for impacted radial head with lateral angulation, avulsed medial collateral ligament (MCL), subluxated/dislocated elbow, and coronoid fragments. Surgical records and/or MRI scans were checked for intact lateral bundles of LUCL, MCL edema, or tear. Plain radiographs were normally taken before and after the emergency reduction. Preoperative radiographs can reveal the radius and ulna dislocations, while the postoperative radiographs can better reveal the impacted radial head fracture, the angulation of the impaction, and the medial coronoid fragment that suggest MCL avulsion. CT slices with 3D reconstruction provide a more detailed evaluation but are mainly useful when the relative position of each fragment is unclear on X-ray. MRI offers additional insights, such as the continuity of the LUCL and asymmetrical bone contusions—typically more extensive in the radial head than in the coronoid. These findings, when combined with fracture morphology, can support a more reliable deduction of the injury mechanism (9). In patients who undergo surgery, intraoperative observation can further confirm the injury pattern.

Radial head fracture classification [Mason classification (3)] along with coronoid process fracture classification [Regan and Morrey classification (1) and O’Driscoll classification (2, 10)] were used to categorize all fractures based on the fracture morphology. The Mason classification considers only the radial head fracture and does not account for elbow dislocation; otherwise, every TTI case would be classified as type IV, since the elbow is dislocated by definition.

A chart review was carried out to investigate the associated injury rates of this special valgus TTI. Associated injuries included ligamentous rupture, a vascular injury requiring repair, and nerve palsies. Radiographs and charts were also reviewed for evidence of a non-union or loss of reduction in the valgus TTI group.

### Treatment

The reduction of the elbow was applied in the emergency room. Neurovascular injury and comorbidities like anti-coagulation or diabetes mellitus therapy were managed, if not excluded, to safeguard the surgery.

Non-operative treatment was considered only when all of the following conditions were met: (1) no radial head impingement during forearm rotation; (2) the concentric relationship between the trochlear groove, the medial lip of the trochlea, and the capitellum was intact; (3) the coronoid fracture was not classified as Regan and Morrey type III; and (4) the elbow remained stable through a range of motion from 30° to 120°. The decision for conservative management was based on a careful assessment of joint stability and fracture pattern. Specifically, conservative management was selected for patients with stable radial head fractures, preserved elbow alignment, and minor coronoid fractures (Regan and Morrey type I). These patients were followed closely with regular radiographs to monitor for signs of instability or non-union.

Surgery began on the lateral side. Through the lateral approach, the condition of the radial head and LUCL were examined and recorded. Radial head reduction and fixation were performed in most cases using cannulated screws, plates, or nails, depending on the fracture pattern and comminution. Although reduction was straightforward in many cases, radial head fixation remains a complex issue in orthopedic literature, with various options including plates, screws, excision, bone grafting, and prosthesis. In this study, most fixations were achieved with cannulated screws or plates, while radial head prostheses were used in two patients with severe comminution and poor bone quality, where fixation was not feasible. In these cases, prosthesis was selected without prior fixation attempts due to insufficient bone stock used. The condition of the LUCL was reconfirmed and recorded.

In cases where a radial head prosthesis was used, we acknowledge that direct comparison with patients who received fixation may not be entirely appropriate, given the differences in surgical approach and long-term outcomes. Nevertheless, both groups were assessed for elbow stability postoperatively, and all patients were followed for an average of 20.2 months to evaluate functional outcomes. It is important to note that while prosthetic replacement is commonly used for severe comminution, it may lead to different biomechanical properties and long-term stability of the elbow compared to fixation.

After radial head fixation, elbow stability was assessed using the gravity-assisted varus/valgus stress test with the elbow flexed at 30° and the forearm in supination. If instability was detected, a medial approach was used to expose the medial aspect of the elbow—either through the soft tissue gap caused by the injury or, if not present, through the interfascial space between the flexor carpi ulnaris and the flexor pronator mass. A suture anchor was used to repair any medial avulsion and/or ligament tears. Elbow braces were applied as needed.

### Rehabilitation and evaluation

Range of motion exercises were encouraged within the first week to build pain tolerance, for both operative and non-operative cases. Clinical assessments were carried out every 2 weeks in the first month and then every 4 weeks in the subsequent 3 months, and then every 3 months within the first year. The Mayo Elbow Performance Score and Index (MEPS and MEPI) and Quick-Disabilities of the Arm, Shoulder, and Hand score (Q-DASH) were used to evaluate elbow function. Heterotopic ossification (HO) was assessed using the Hastings classification (11). Follow-up data before July 2020 were analyzed.

### Statistics

All analyses were performed using the Statistical Package for the Social Sciences version 24.0 (IBM Corp., Armonk, NY, USA). Continuous variables were compared using independent-sample *t*-tests (two-tailed). Categorical variables were compared using Pearson's chi-square tests; however, if any expected cell count was below 5 (due to imbalanced group sizes or rare events), Fisher's exact test was used instead to ensure statistical validity. Statistical significance was set at *p* < 0.05 for all analyses. Before conducting the *t*-test, we performed the Shapiro–Wilk test to assess whether the data followed a normal distribution. We also visually examined the distribution using histograms and Q-Q plots to further support our assessment. After confirming that the data followed a normal distribution (as indicated by the normality tests and visual inspection), we opted to use the independent samples *t*-test to compare the means of the two groups. The kappa coefficient was used to analyze the inter- and intra-observer reliability of classification agreements.

## Results

### Demographics

Of the 313 patients with elbow dislocations, valgus-type TTI was identified in 13 (4.2%) patients with a minimum follow-up of 12 months (Table 1). This group included eight women and five men, with a mean age of 45.8 years—nearly half (six cases) were aged over 50 years. The remaining 300 patients had a mean age of 40.9 years, though the difference was not statistically significant (*p* = 0.2). Low-energy trauma was the main cause (11 of 13, 84.6%), with falls accounting for the most common mechanism of injury (10 of 13, 76.9%). No neurovascular injuries were observed in the valgus-type TTI group.

**Table 1:** Characteristics of the study population and final functional results.

No.	Age (years)	Gender	Injury mechanism	Radial head fracture type	Coronoid process fracture type (Regan and Morrey/O'Driscoll)	Fracture treatment	Soft tissue treatment	Follow-up (months)	Postoperative ROM at 1 year (F/E; P/S)	Q-DASH	MEPS	Complication
1	70	Female	Fall	Mason III	I/Anteromedial sub 3	Brace	None	24	105°/25°; 85°/85°	6.8	100	HO(I), MCL avulsion non-union, RH malunion
2	68	Female	Fall	Mason II	I/Anteromedial sub 3	Screw	None	36	142°/0°; 85°/90°	0	100	—
3	23	Female	Fall	Mason II	I/Anteromedial sub 3	Screw	Suture anchor	23	130°/0°; 75°/90°	4.5	100	MCL avulsion non-union
4	35	Male	Traffic accident (motorcycle)	Mason II	I/Anteromedial sub 3	Screw	None	30	120°/10°; 80°/90°	6.8	100	—
5	16	Male	Traffic accident (bike)	Mason II	I/Anteromedial sub 3	Screw	Suture anchor	19	135°/0°; 85°/85°	4.5	100	—
6	44	Female	Fall	Mason III	I/Anteromedial sub 3	Brace	None	18	115°/0°; 70°/85°	4.5	100	HO(IIC), MCL avulsion non-union, RH malunion
7	31	Female	Fall	Mason III	I/Anteromedial sub 3	Brace	None	16	120°/15°; 80°/85°	12.5	100	HO(IIA), MCL avulsion non-union, RH malunion
8	55	Male	Fall	Mason II	I/Anteromedial sub 2	Screw	Suture anchor	22	125°/10°; 85°/85°	4.5	100	—
9	43	Male	Fall	Mason III	I/Anteromedial sub 3	Arthroplasty	None	18	140°/10°; 85°/75°	11.4	100	—
10	28	Female	Traffic accident (motorcycle)	Mason III	I/Anteromedial sub 2	Arthroplasty	None	15	140°/0°; 85°/90°	0	100	MCL avulsion non-union
11	64	Female	Fall	Mason II	I/Anteromedial sub 2	Nail	None	14	135°/10°; 85°/75°	0	100	MCL avulsion non-union
12	52	Female	Fall	Mason II	I/Anteromedial sub 2	Plate and screw	Suture anchor	15	120°/0°; 85°/80°	2.5	100	HO(IIA), MCL avulsion non-union
13	66	Male	Fall	Mason II	I/Anteromedial sub 3	Screw	None	12	125°/15°; 85°/85°	25	85	MCL avulsion non-union
Mean ± SD	—	—	—	—	—	—	—	—	127.08° ± 11.12°/7.31° ± 8.07°/82.31° ± 4.84°/84.62° ± 5.19°	6.38 ± 6.84	98.85 ± 4.16	—

F/E, flexion/extension; P/S, pronation/supination; Q-DASH, Quick-Disabilities of the Arm, Shoulder, and Hand score; MEPS, the Mayo Elbow Performance Score; ROM, range of motion.

### Injury patterns

**Figure 1:**
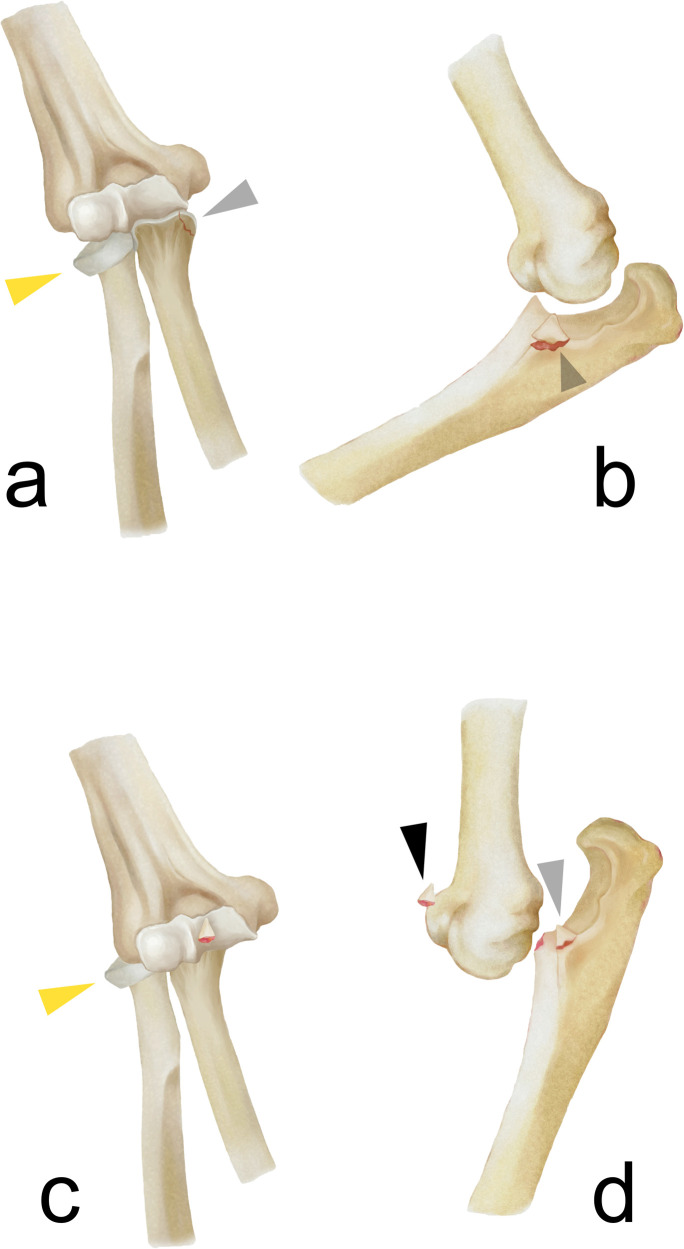
The fracture pattern of the valgus-type TTI with the lateral angulated radial head (yellow triangle), MCL avulsion (gray triangle), and posterolateral dislocation/subluxation **(a,b)**. Theoretically, if the injury force is substantial, the elbow dislocation can be more severe **(c)**, resulting in an additional coronoid tip fragment (black triangle) and producing an injury pattern similar to non-valgus TTIs **(d)**.

All valgus TTI patients were observed to have posterolateral elbow dislocation rather than a posterior dislocation (Figure 1). All radial head fractures were minimally displaced with lateral angulation; one-third were comminuted. The coronoid fragments were all located at the anteromedial facet (O’Driscoll type II) and measured 14.5% ± 2.0 of the total coronoid height (Regan and Morrey type I).

Inter-observer agreement was moderate across all fracture classification systems, with Cohen's κ values of 0.55 for the Mason classification (radial head fractures), 0.50 for the Regan–Morrey classification (coronoid fractures), and 0.60 for the O’Driscoll classification, corresponding to moderate to substantial agreement by standard criteria. Intra-observer reliability was higher for each system, with *κ* values of approximately 0.70 for Mason, 0.65 for Regan–Morrey, and 0.75 for O’Driscoll.

### Surgical findings

Three patients were treated non-operatively using elbow braces, while the remaining 10 patients underwent surgery. Radial head fixation was performed with screws in seven patients, nail fixation in one, and radial head arthroplasty in two. Intraoperative examination confirmed that the LUCL was intact in all cases. One patient with a significant dislocation had a partial LUCL tear (sprain grade II) without loss of continuity; the others had grade I sprains but intact ligaments. All coronoid fractures resulted from avulsion of the anterior bundle of the MCL, consistent with preoperative radiographs. Joint instability was observed in four patients after radial head fixation, and suture anchors were used to repair their MCL avulsions.

### Outcomes

With the above treatment strategy, no re-operation was applied after the initial treatment. The mean follow-up was 20.2 months (range 12–36 months). At the last follow-up, the mean MEPS was 98 (range 85–100) and the mean Q-DASH score was 6.38 (range 0–12.5). The range of elbow flexion, extension, pronation, and supination were 127.1° ± 10.7, 7.3° ± 7.7, 82.3° ± 4.6, and 84.6° ± 5.0, respectively. Non-union was quite common with MCL avulsion fragments, with over half (8/13) of the patients experiencing non-union without affecting elbow stability and range of motion. The remaining patients experienced radiographic union at a mean of 10.7 weeks. HO was observed in four patients. No neuropathy was observed in this group of patients (Table 1).

### Case example

#### Case 1

**Figure 2:**
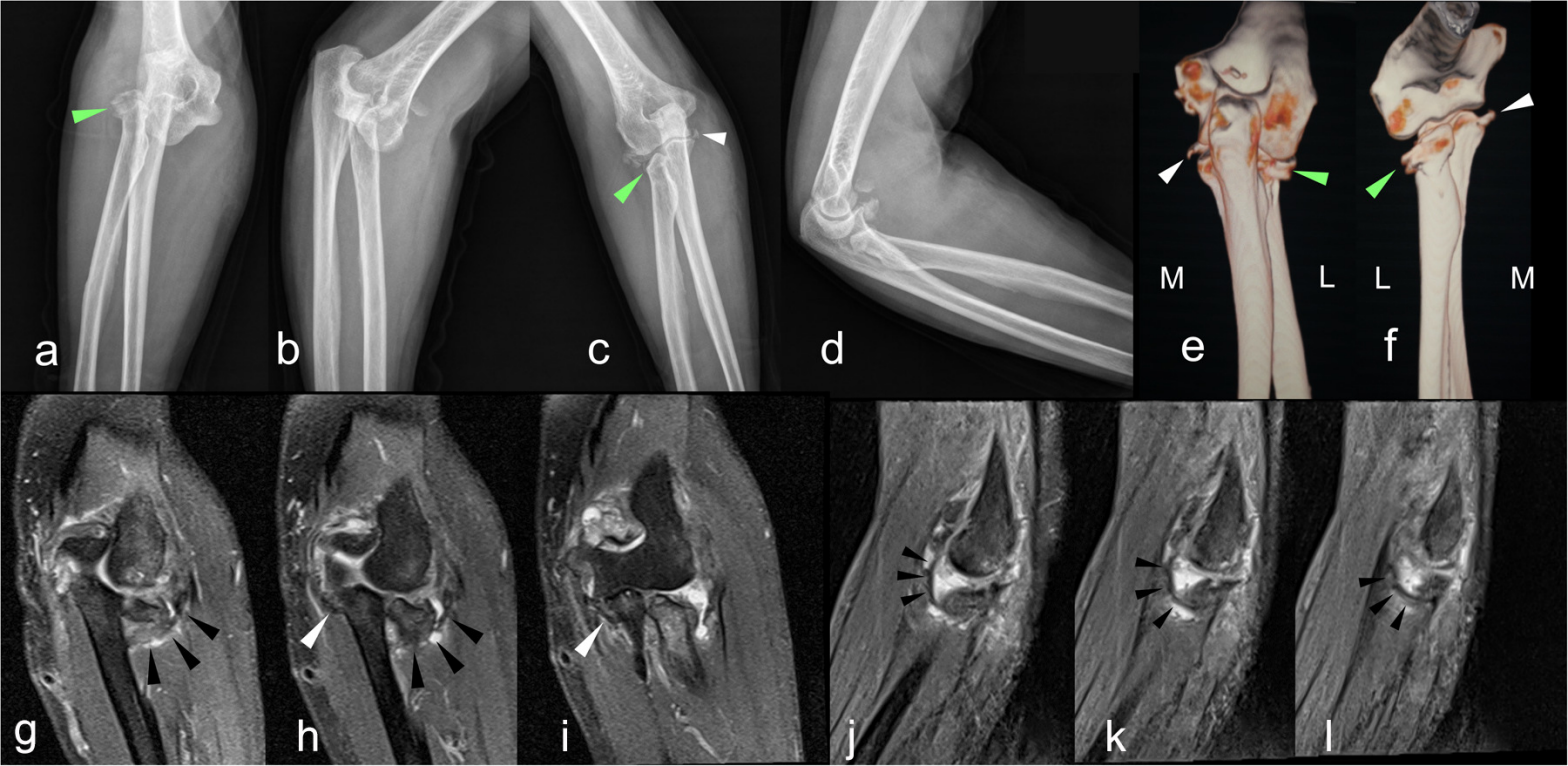
A 70-year-old woman fell on her extended left arm (case 1). Radiographs demonstrated a posterolateral dislocation **(a,b)**; after the reduction **(c,d)**, the laterally angulated radial head impaction and coronoid fragment can be found. **(e,f)** The back and front view of the forearm bones on 3D CT show the laterally impacted radial head (green triangles) and the avulsed coronoid fragment (white triangles), located at the anteromedial tip of the coronoid process. Continuous coronal **(g–i)** and sagittal **(j–l)** MRI slices confirmed the continuity of the LUCL (black triangles) and the MCL-related coronoid avulsion. In sagittal MRI views, distinguishing between the LUCL and annular ligament was sometimes difficult. Bone contusion of the radial head was more extensive than that of the coronoid, which was limited to the area surrounding the avulsion. These findings suggest a valgus mechanism. L, lateral; M, medial.

A 70-year-old woman fell on her extended left arm. A radiograph demonstrated posterolateral dislocation. After the reduction, the laterally angulated radial head impaction and coronoid fragment can be found in the postoperative radiograph. The laterally impacted radial head and the avulsed coronoid fragment can be identified on 3D CT. The avulsion was located at the anteromedial tip of the coronoid process. Continuous coronal and sagittal MRI slices confirmed the continuity of LUCL and the coronoid avulsion of MCL. Sometimes it was difficult to distinguish between the LUCL and the annular ligament in the sagittal view of MRI. Bone contusion of the radial head was more extensive than that of the coronoid, where the contusion was confined only to the near area of the avulsion. These findings suggest a valgus mechanism (Figure 2).

**Figure 3:**
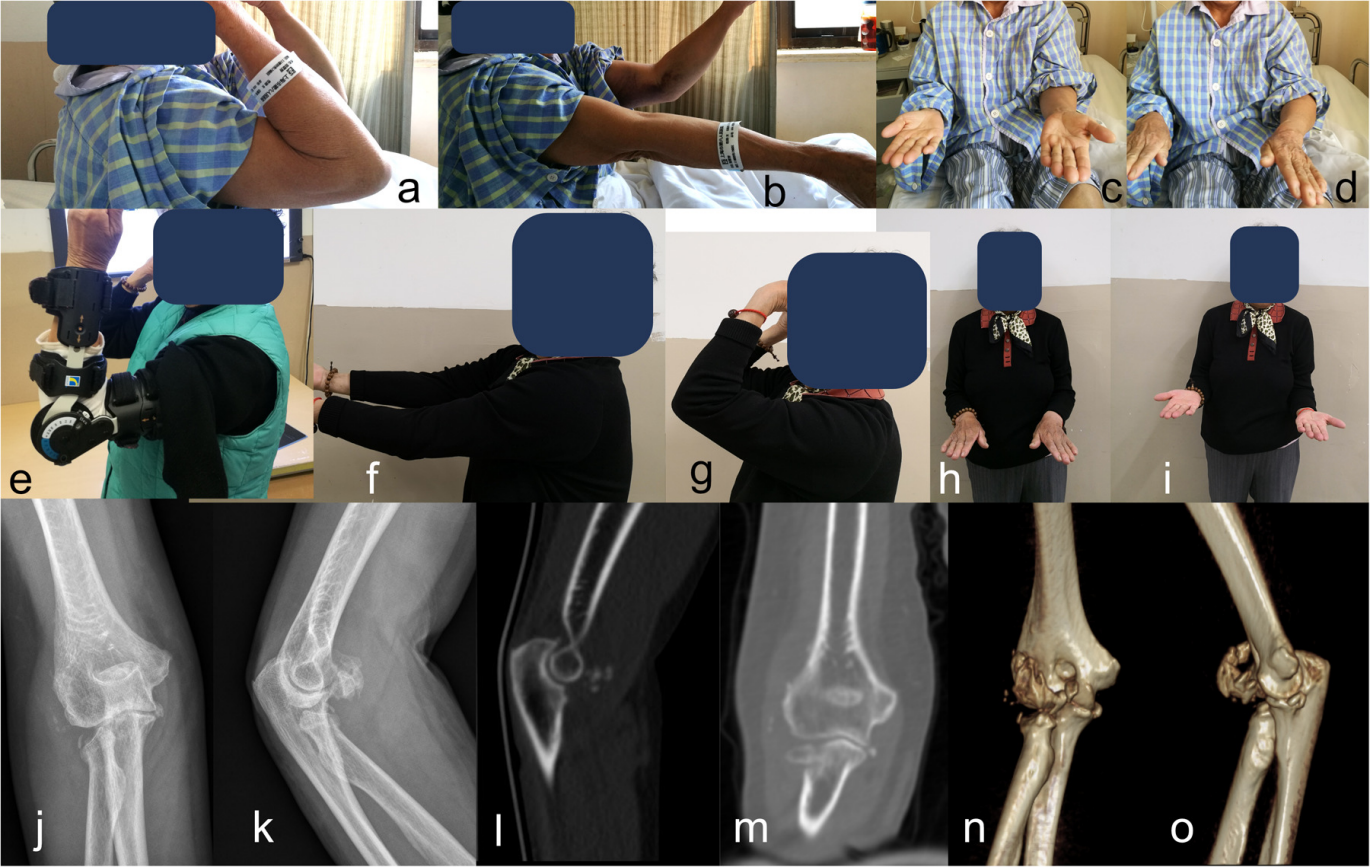
Non-operative treatment of the patient whose fracture patterns are shown in Figure 1. Elbow extension and pronation were limited after the injury **(a–d)**. Though dislocated, the elbow joint instability was relatively easy to regain after reduction in the valgus-type TTI; elbow brace was used to facilitate rehabilitation **(e)**. Joint function was improved after 12 months **(f–i)**; radiographs show the radial head malunion, non-union of MCL avulsion, and lateral heterotopic ossification **(j–o)**.

Figure 3 shows the non-operative treatment of the patient whose fracture pattern is shown in Figure 1. Elbow extension and pronation were limited after the injury. Although dislocated, joint instability in valgus-type TTI was relatively easy to restore after reduction, and an elbow brace was used to support rehabilitation. Joint function improved after 12 months. Radiographs showed radial head malunion, non-union of the MCL avulsion, and lateral heterotopic ossification (Figure 3).

#### Case 2

**Figure 4:**
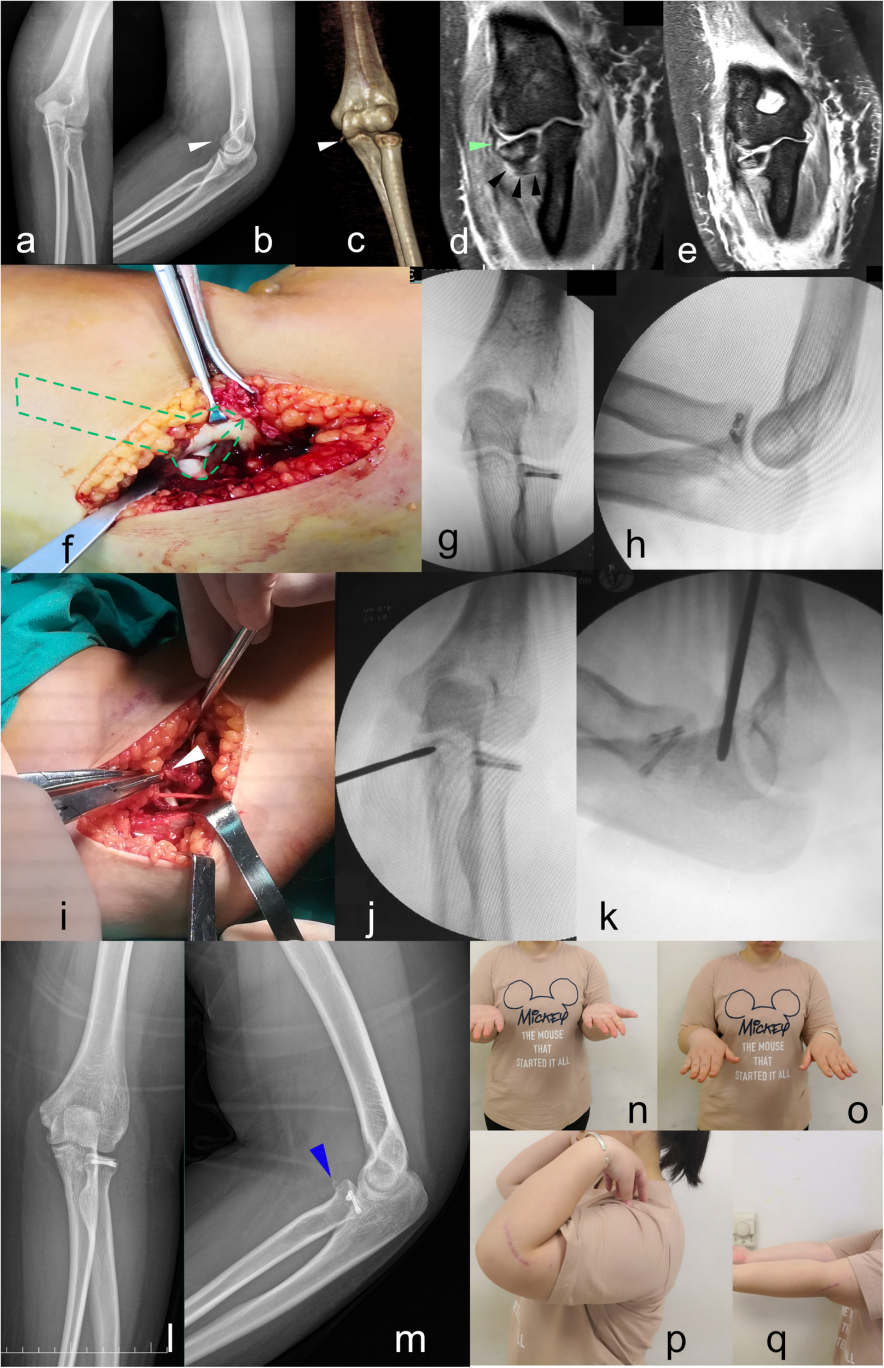
A 23-year-old woman (case 2) with valgus-type TTI. Dislocation history was reported by the patient. Preoperative radiographs **(a–e)** showed lateral angulation of the radial head, medial avulsed fragment (white triangle), intact LUCL (black triangle), and intact radial collateral ligament (green triangle). Bone contusion was found less extensive in the medial half of the elbow joint **(e)**. Joint instability only recurred under valgus stress. Intraoperatively, the radial head was fixed by two screws **(f–h)**. The anterior bundle of the MCL had avulsed and was repaired by a suture anchor **(i–k)**. At the 1-year follow-up, the MCL avulsion non-union (blue triangle) did not affect joint function **(n–q)**.

A 23-year-old woman with valgus-type TTI had a self-reported history of dislocation. Preoperative radiographs showed lateral angulation of the radial head, medial avulsed fragment, intact LUCL, and intact radial collateral ligament. Bone contusion was less extensive in the medial half of the elbow joint. Joint instability only recurred under valgus stress. During the operation, the radial head was fixed with two screws, and the anterior bundle of the MCL was found avulsed and repaired using a suture anchor. At the 1-year follow-up, the MCL avulsion non-union did not affect joint function (Figure 4).

## Discussion

This study provides a detailed analysis of a rare and unique injury pattern of TTI of the elbow resulting from a valgus mechanism. Our findings show that valgus-type TTIs exhibit distinct radiographic and surgical features, including radial head fractures with lateral angulation, coronoid avulsion at the origin of the MCL, and an intact LUCL. This injury pattern, which has received limited attention in the literature, calls for a tailored treatment strategy that emphasizes early identification of instability and selective repair of MCL avulsion. Recent studies have highlighted the need for individualized treatment in TTI cases, acknowledging that while standard protocols are useful, variations in injury patterns demand customized approaches to optimize outcomes (12–18). The most important finding of this study is that valgus-type TTIs, despite involving significant elbow injury, can achieve good functional outcomes with appropriate management—whether conservative or surgical –based on the specific injury pattern.

Fracture classification systems for complex elbow injuries help guide surgeons in identifying associated injuries and planning appropriate treatment. The O’Driscoll classification is currently the most comprehensive and widely accepted for understanding TTI, describing the sequential injury of peripheral bony and ligamentous structures. Type I injuries, characterized by PLRI, account for 92% of TTIs and typically begin with LUCL injury. However, the injury pattern identified in our study deviates from this classic sequence, despite meeting the criteria for TTI. Less common types (II and III, representing 7% and 1%, respectively) also differ significantly from the valgus mechanism we observed. Morphologically, the fractures and associated injuries observed suggest a less severe, valgus-related trauma, offering novel insights into the current understanding of TTIs.

Accurate diagnosis and understanding of the injury pattern facilitate optimal surgical planning. Multiplanar radiographs and CT scans, particularly with 3D reconstructions, effectively identify subtle injury details. In cases caused by valgus force, radial head impaction with lateral displacement is evident. MRI can confirm partial tears of the MCL and LUCL, although a detailed CT is usually sufficient. During open reduction, ligament-attached avulsion fragments can typically be identified, consistent with posterolateral elbow dislocation.

The mechanism of elbow instability has always been controversial. Although PLRI remains a well-accepted model of elbow instability, other findings suggest that the sequence of soft tissue disruption may begin medially. A recent videographic study suggested that acute elbow dislocation may result from a gross valgus deforming moment with subtle external rotation (14). Biomechanically, the injury is more likely begin at the anterior bundle of the MCL, as it is the primary soft tissue restraint again valgus force. In the cases we observed with a similar mechanism, the elbow joint was subluxated, with an impacted radial head fragment angulated laterally and an associated MCL avulsion—features consistent with a valgus deforming tendency at the elbow. The LUCL may sustain a grade I sprain but remains intact. A similar valgus-like injury pattern was previously reported by Cho et al. (15), involving a radial head fracture with associated MCL injury. In their case, the radial head fragment was facing obliquely to the lateral side, indicating a major valgus force. However, this injury pattern did not involve elbow dislocation or coronoid fracture, resulting in relatively minor soft tissue compromise and less elbow instability. Therefore, it did not meet the diagnostic criteria for TTI. In addition, another valgus-like injury was also noticed by Rhyou et al., which combines the radial head injury and the coronoid process (9). Although there was no elbow dislocation, it still proves that the valgus mechanism plays a role in the elbow injury. Based on these findings, we speculate that a valgus force can lead to a series of injuries, depending on the energy involved. The elbow may be subluxated, fully dislocated, or remain aligned. When both elbow subluxation and an anteromedial coronoid fragment are present (Figures 4a,b), the injury meets the diagnostic criteria for TTI. Furthermore, when the deforming force is greater, the elbow may become fully dislocated, shearing off the tip of the coronoid process and creating an additional coronoid tip fragment (Figures 4c,d). In such cases, it becomes harder to differentiate this injury from non-valgus TTIs.

Still, two facts are noteworthy in this study. First, based on our surgical experience, patients in this group appeared to have relatively poor bone quality. They were mostly inactive elderly or younger patients, both of whom may be more prone to fracture from lower-energy trauma, although this was not assessed. Second, the dislocation direction in valgus TTI cases was posterolateral rather than purely posterior—this direction may contribute to joint instability and could provide insight into the injury mechanism. However, further research is needed to reliably explain these observations.

Several strategies have been described for treating valgus TTI that addresses its key injury components. The treatment strategy for valgus-type TTI focuses on the unique injury pattern caused by the valgus mechanism, characterized by compression fractures of the radial head and avulsion of the MCL, while the LUCL remains intact. This biomechanical profile differs from the classic TTI, which typically involves PLRI with initial LUCL tear, followed by radial head and coronoid fractures. In our study, the treatment strategy begins with a lateral approach to assess and fix the radial head. This step is common to all TTIs but is followed by a careful evaluation of elbow stability under varus/valgus stress. If instability is detected, a medial approach is used to repair the avulsed MCL. This approach differs from standard TTI management by specifically addressing the unique medial injury of the MCL without compromising the intact LUCL. The valgus mechanism results in a more stable elbow joint after reduction compared to the classic PLRI. Therefore, our treatment strategy emphasizes individualized assessment of joint stability rather than a blanket fixation approach for all components. Overall, the treatment is relatively simpler than that of classic TTIs. Reduction and fixation start at the radial head; meanwhile, the continuity of the LUCL is confirmed. Elbow stability is then tested, and a positive finding indicates the need for surgical repair of the medial structures. Early mobilization begins as pain allows after the operation, with a motion range-adjustable elbow brace if needed. The described techniques yielded successful outcomes both radiographically and clinically in all presented cases.

In our series, the mean final MEPS was 95 points (excellent) and the mean Q-DASH score was 10, reflecting very favorable elbow function. These outcomes are comparable to those reported for classic terrible triad injuries in the literature. For example, a recent scoping review of modern TTI cases found a mean MEPS of approximately 90 (excellent) and a mean Q-DASH score of 13 for surgically managed patients, indicating generally good to excellent results across studies (19). Similarly, Giannicola et al. reported a mean MEPS of 96 with a Q-DASH score of 8 in a prospective series of classic TTIs. Compared to these benchmarks, our cohort's outcomes are on par with the best reported results for classic TTI, underscoring that patients with this valgus mechanism injury pattern can achieve excellent functional recovery with appropriate management.

The present study has some limitations. First, the valgus mechanism is an empirical judgment based on the radiographic findings and surgical findings; therefore, more evidence is surely required to substantiate it. Second, we acknowledge that the sample size of the valgus mechanism TTI cohort was necessarily small (*n* = 13) due to the rarity of this injury mechanism, which in turn limits the statistical power of comparisons with the non-valgus TTI group. Consequently, any differences observed between the valgus and non-valgus TTI cohorts should be interpreted with caution, and our comparative findings regarded as descriptive in nature, given this limitation. In addition, although we aimed to simplify treatment strategies, the fixation constructs varied between cases, so the approaches described cannot be fully validated by this study. Lastly, due to the exploratory nature of this study and its small sample size, we did not apply formal correction for multiple testing. We acknowledge that this increases the risk of a type I error (false-positive result), and therefore the results should be interpreted with appropriate caution.

## Conclusion

The valgus-type TTI represents a unique pattern within complex elbow injuries. Its radiographic hallmarks include the following: (1) radial head fracture with lateral angulation, (2) intact LUCL, (3) coronoid avulsion of MCL, and (4) posterolateral elbow subluxation or dislocation. Surgeons should be aware of how this pattern differs from traditional TTIs. The reduction and fixation strategies described can be used to manage this injury. Functional outcomes appear similar to those reported for classic TTIs in the literature.

## Data Availability

The raw data supporting the conclusions of this article will be made available by the authors, without undue reservation.
